# Supplementary figures and images for: Non-markovian electron tunneling in SARS-CoV-2 virus infection in structured environments
Source: PLoS One. 2026 Apr 3;21(4):e0344447. doi: 10.1371/journal.pone.0344447 (PMC13048415; doi:10.1371/journal.pone.0344447)

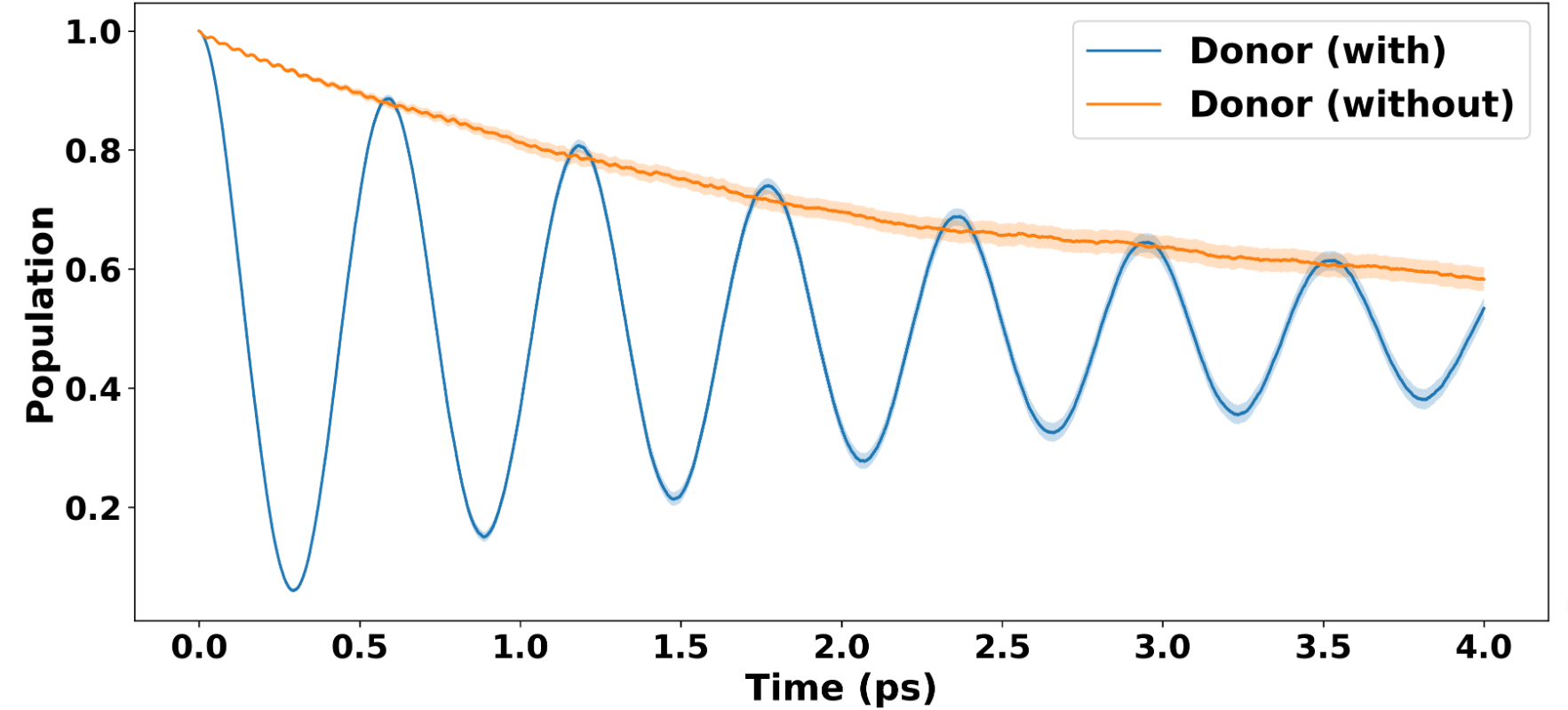

Supplement: S1 Fig — Note: All statistical analyses are presented in the Supporting Information (SI). (PNG) [file pone.0344447.s001.png]

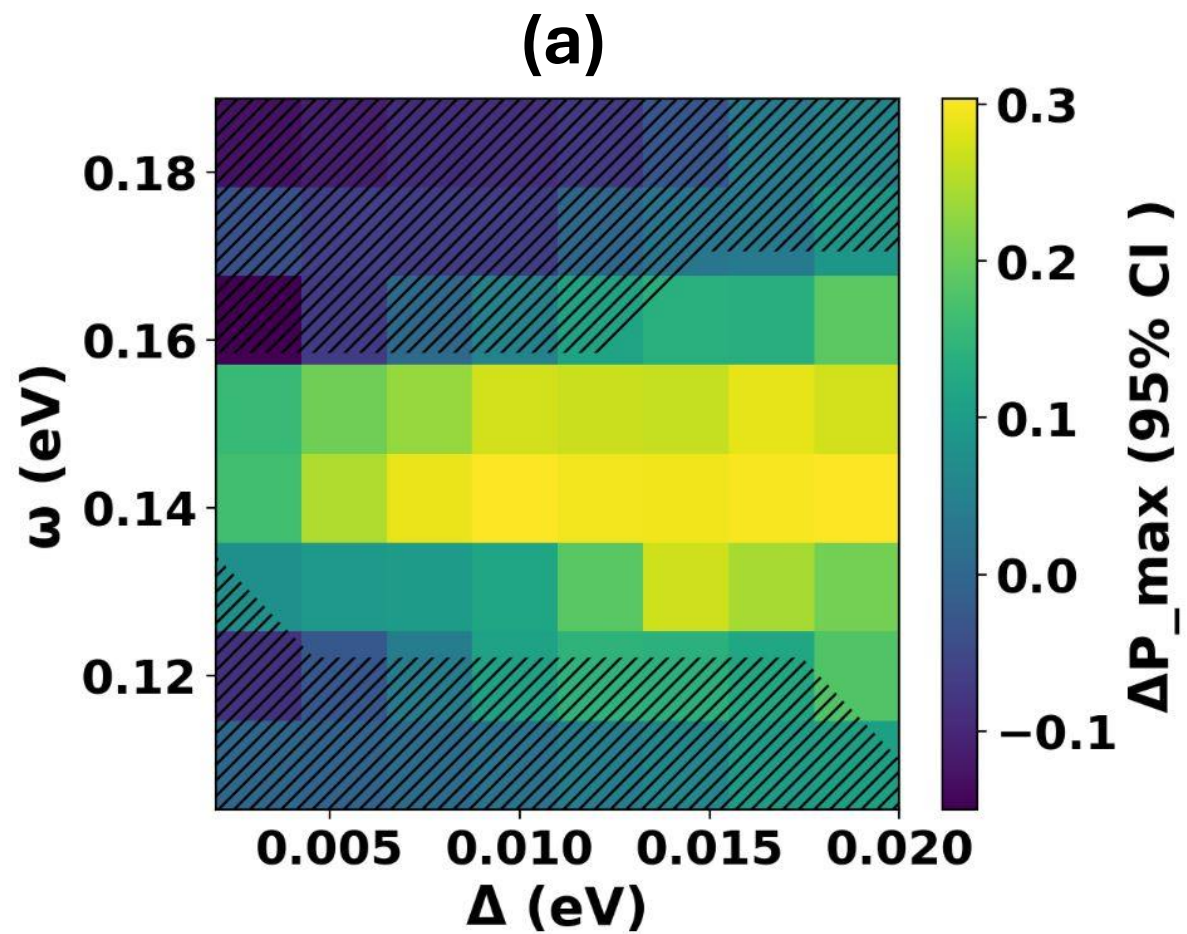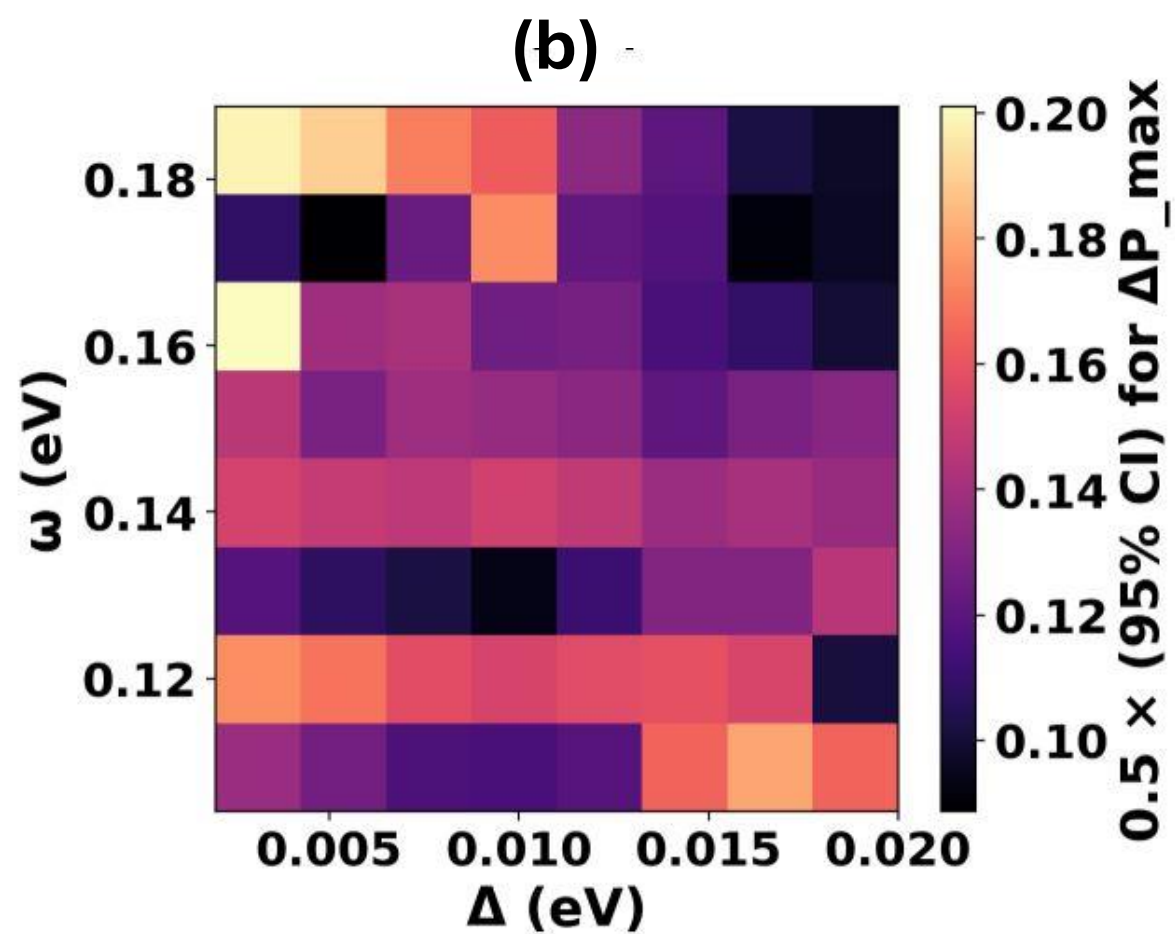

Supplement: S2 Fig — (PDF) [file pone.0344447.s002.pdf]

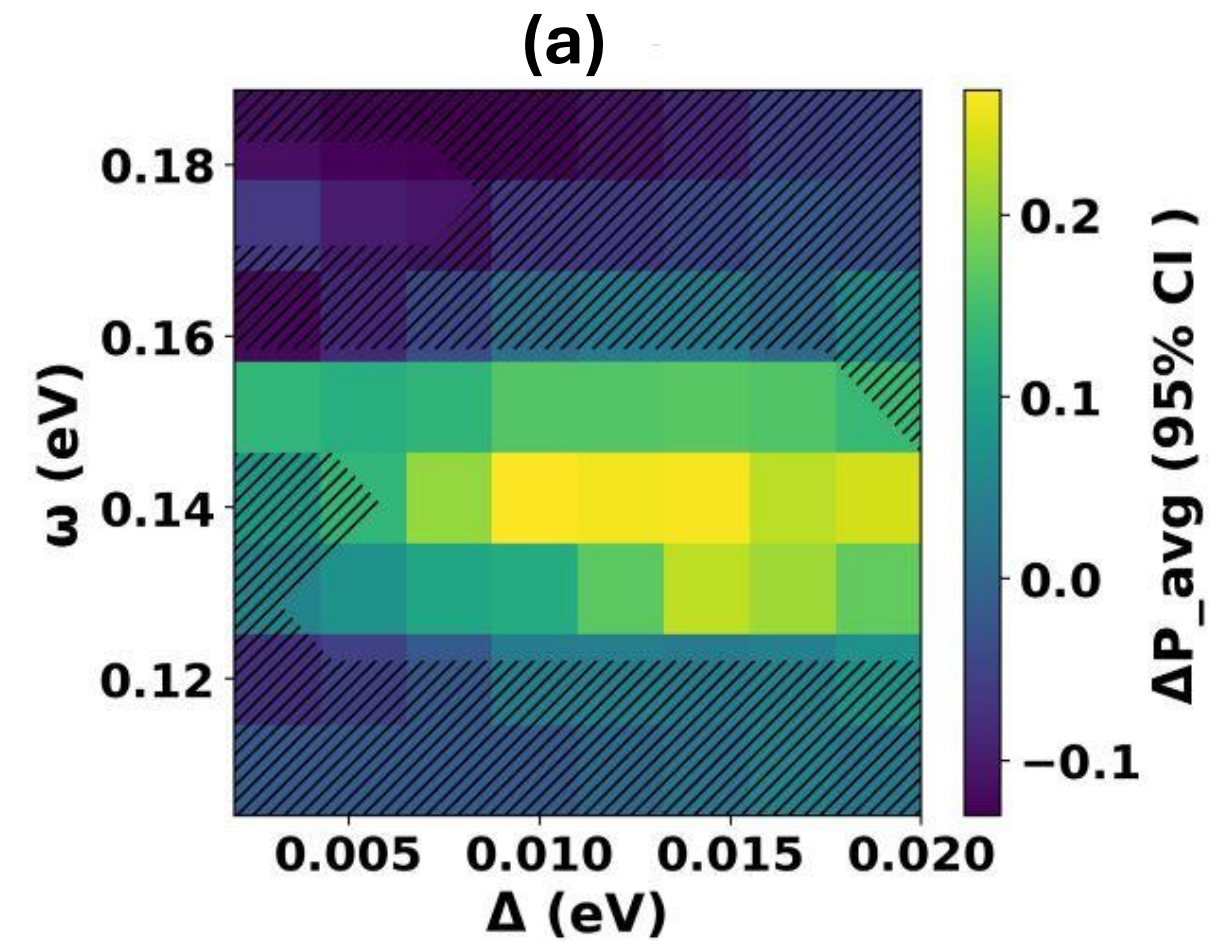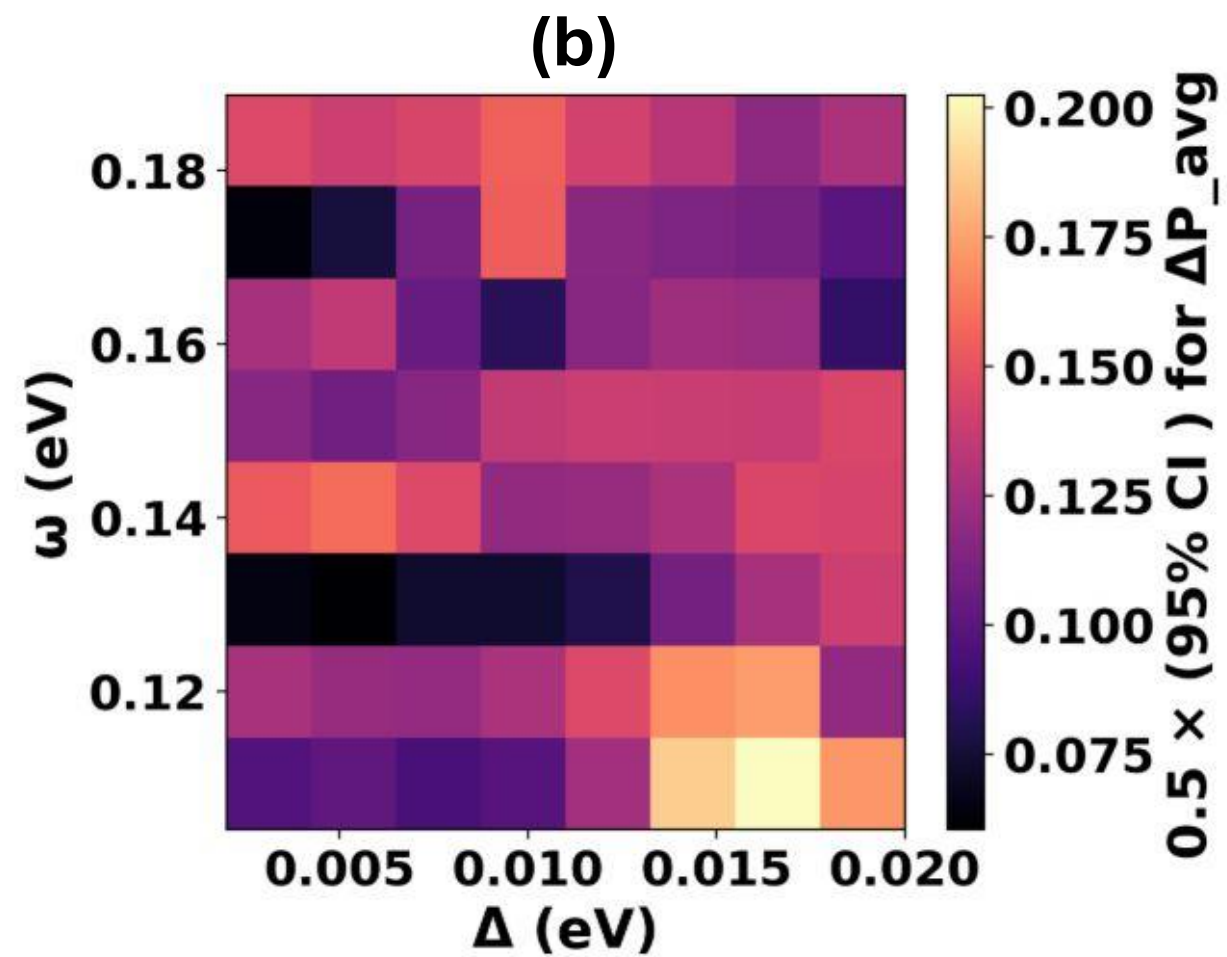

Supplement: S3 Fig — (PDF) [file pone.0344447.s003.pdf]

**(a)**

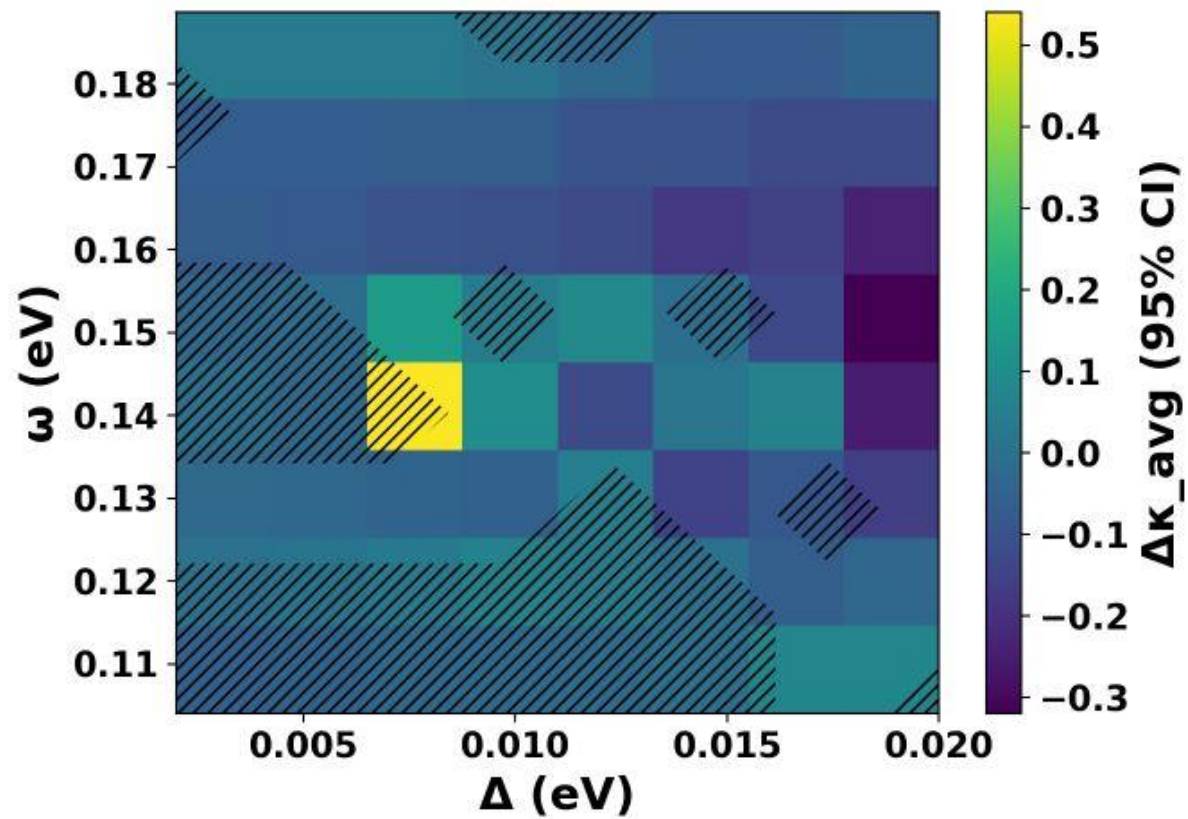

**(b)**

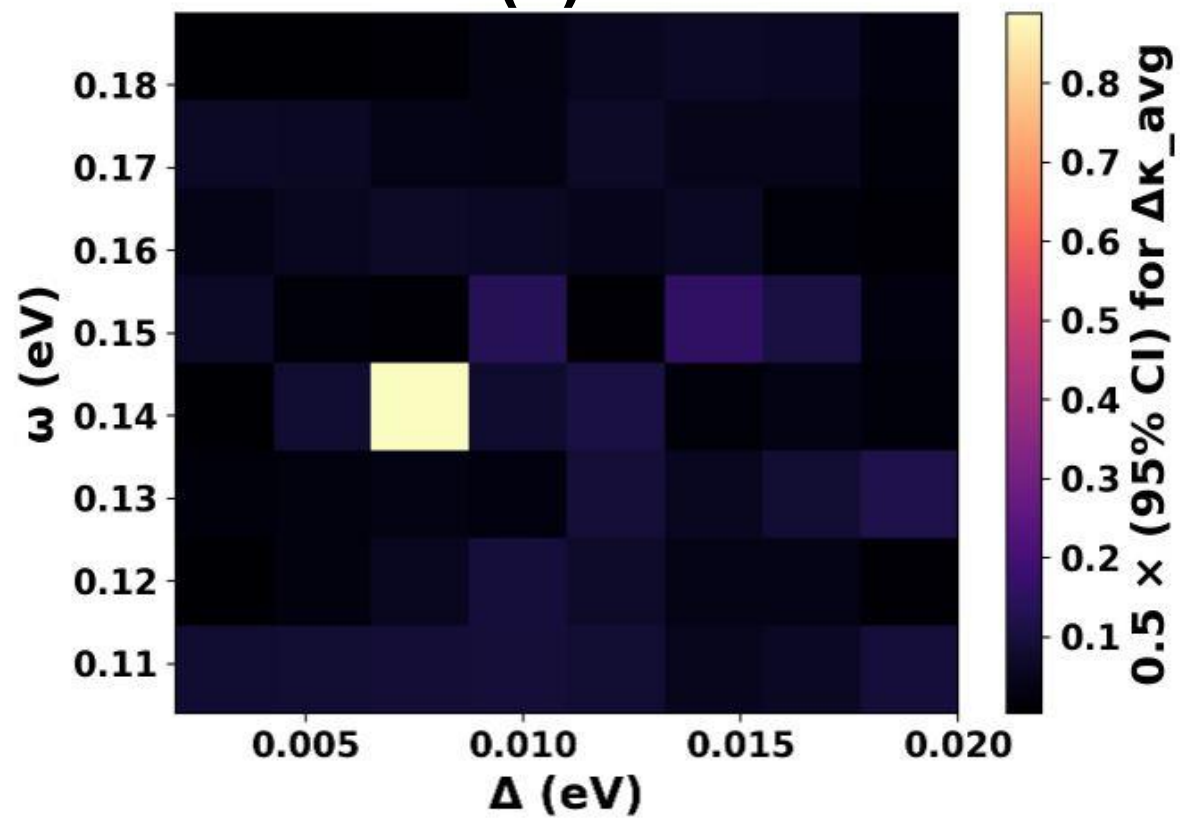

Supplement: S4 Fig — (PDF) [file pone.0344447.s004.pdf]
