# Supplementary material for: Non-markovian electron tunneling in SARS-CoV-2 virus infection in structured environments
Source: PLoS One. 2026 Apr 3;21(4):e0344447. doi: 10.1371/journal.pone.0344447 (PMC13048415; doi:10.1371/journal.pone.0344447)
Supplement: Attachment — Submitted filename: Response_letter_PLOS.pdf [file pone.0344447.s005.pdf]

Response to Reviewers

Submission ID:[PONE-D-25-45999] - [EMID:88623151bdfdb171]

Manuscript Tittle: Non-Markovian Electron Tunneling in SARS-CoV-2 virus infection in Structured Environments.

Dear Editor and Reviewers,

We thank you for the careful reading and constructive suggestions. We have revised the manuscript, figures, and code accordingly. Below we respond point-by-point, quoting each comment in bold and then providing our response. Where helpful, we include exact text to be inserted into the manuscript and note figure/code changes.
Reviewer #1 :

We would like to express our sincere gratitude for your thorough review and insightful comments on our manuscript. Your feedback has been invaluable in guiding us to refine and enhance our study, ensuring that the final version of our paper is both robust and comprehensive. In response to your observations and suggestions, we have carefully revised our references.

In the following sections of this response letter, we detail the modifications in the references and additions we have made to our manuscript in light of your feedback.

References 1–3, 10–17, 20, 21, 25, 28, 29, 31–33, 35, 36, are old, please use new references.

Response: We thank the reviewer for this valuable suggestion. We have systematically updated the bibliography throughout the manuscript. All items flagged as "old" (Refs. 1–3, 10–17, 20, 21, 25, 28, 29, 31–33, 35, 36) were reviewed and, where appropriate, *replaced with recent peer-reviewed sources* from the last few years. We also *added contemporary reviews and primary research articles* to reflect current advances in non-Markovian open-quantum dynamics, vibronic electron transfer, and structured (protein) environments relevant to SARS-CoV-2. A small number of classic citations were retained because they are the seminal sources of widely used formalisms; these are now explicitly identified as foundational in the text.

All updates are visible in the revised reference list and highlighted in orange color in the tracked-changes version.

Reviewer #2

We would like to express our sincere gratitude for your thorough review and insightful comments on our manuscript. Your feedback has been invaluable in guiding us to refine and enhance our study, ensuring that the final version of our paper is both robust and comprehensive. In response to your observations and suggestions, we have carefully revised our manuscript, addressing each point you raised to clarify our methodology, strengthen our analysis, and better articulate our findings.

In the following sections of this response letter, we detail the modifications and additions we have made to our manuscript in light of your feedback. For each comment or concern you expressed, we provide a summary of the changes implemented, offering explanations where necessary to elucidate how these adjustments contribute to the overall quality of our research. Furthermore, we have created the supporting information(SI) to present all the statistical analysis and comments you raised in the review. We believe that these revisions have significantly improved our paper, making it a more valuable contribution to the field.

1) Technical Soundness and Data Support

1.1 Methods, Lines 110–150: Numerical implementation details

"Numerical implementation details are missing. Please specify the integrator used, time step, ensemble size (number of trajectories), and convergence checks. Without this, the reproducibility of results is unclear."

Response: We added a dedicated **Numerical Implementation** paragraph in **Numerical Results** specifying the integrator, step size, trajectory counts, seeding, and convergence. Inserted text:

We Solve (Eq.26) with an Euler–Maruyama (Itô) scheme. For the state $\psi(t)$ we solve with Lindblad operator $L = \gamma_E \sigma_z$, Gaussian Wiener increment $dW_t \sim \mathcal{N}(0, dt)$, and Hermitian H as defined in Eqs. (1–11). Unless otherwise noted we use a fixed time step $\Delta t = 0.005$ and total propagation time $T = 4\,\text{ps}$. Observables are computed as ensemble means over $N = 1000 - 2000$ independent trajectories per condition; in two-condition comparisons we employ a paired design (the same random seed per trajectory across conditions) to reduce variance. Uncertainty is reported as mean $\pm$ 95% confidence intervals obtained by nonparametric bootstrap over trajectories (typically $B = 3000$ for time series and $B = 5000$ for parameter-sweep heat maps). Numerical convergence is verified by halving the time step ($\Delta t/2$) and reporting the relative change in terminal donor survival population; in all figures this change is $< 2\%$ (the exact value is listed in each caption). Random numbers are generated with a fixed seed range $\{s_0, \ldots, s_0 + N - 1\}$ for full reproducibility, and the simulation scripts output the exact Δt, T, N, seeds, and Hamiltonian parameters used.

This text is highlighted in the Numerical Results Section in orange color.

1.2 Figure 3, Lines 250–300: Add uncertainty

"Population transfer is shown as color plots, but no error bars or variance measures are provided. Please include uncertainty quantification."

Response: We now provide **uncertainty quantification** in Supproting information:

- For 1D time-series (donor survival population(ρ_{00}): **mean $\pm$ 95% bootstrap CIs** (trajectory bootstrap; $B = 3000$).
- For heat maps (parameter sweeps over Δ and ω): a **mean map** and a **CI half-width map**, using **paired** bootstrap with $B = 5000$ per pixel.

1.3 Results, Figure 5, Lines 370–400: Wording

"The phrasing ' particularly with the virus' is grammatically incorrect and unclear; revise to 'particularly with the virus. "
Response: Corrected. The sentence now reads: **"...particularly with the virus."**

1.4 Discussion, Figures 7–8, Lines 420–460: Sensitivity to frequency

"The role of resonance and off-resonance vibrational modes is asserted, but no sensitivity tests are shown. Please demonstrate robustness to small frequency shifts."

Response: We added a **frequency-sweep sensitivity analysis** (new Fig. S1-S3 in SI):

- **Grid:** $\Delta \in [0.002, 0.02]$ eV $\times$ $\omega \in [0.1040, 0.1887]$ eV.
- **Per-pixel:** $N = 1000 - 2000$ paired trajectories, $\Delta t = 0.005$, $T = 4$ ps.

- **Observables:** ΔP (difference of peak acceptor populations) and time-averaged ΔP_{avg} over $[0.5\text{ ps}, 4\text{ ps}]$.
- **Uncertainty:** paired bootstrap CIs ($B = 5000$) per pixel.

We observe maximal enhancement near resonance and smooth decay off-resonance; CI-width maps demonstrate robustness.

2) Statistical Analysis

2.1 Methods, Lines 90–100: Report N and error analysis

"The manuscript notes 'ensemble-averaged density matrix' but does not report the number of trajectories (N) used. Please add this information and provide an error analysis."

Response: :We now report N **explicitly** in Numerical Results and Discusion Section, and added an **Error Analysis** paragraph in the SI Information:

> **Error Analysis and Confidence Intervals.** All time-series are shown as trajectory **means** with **95% bootstrap CIs** ($B = 3000$). For between-condition differences (e.g., ΔP) we use a **paired design** (shared random seeds), compute per-trajectory differences, and report the mean with 95% CIs from resampling those differences. Parameter-sweep heat maps use $B = 5000$ paired bootstrap resamples per pixel.

2.2 Lines 300–320: CIs for ΔP and κ

"These observables should be reported with confidence intervals or error bars, since they are derived from stochastic simulations."

Response: We now report CIs for both:

- ΔP: paired per-trajectory peak differences with mean and **95% CI**.
- $\kappa(t)$: instantaneous rate from Population (centered finite differences), shown as **mean** $\pm$ **95% CI**.

3) Data Availability

"The current text states, 'data available upon justified request.' This does not comply with PLOS ONE policy. Please deposit all underlying data (raw simulation outputs, processed data behind figures, and ideally code) in a public repository and update the statement accordingly."

Response: We updated the Data Availability Statement. All , **configuration files**, **figure-source data** (time arrays, mean curves, CI bands, sweep summaries), and **run metadata** are deposited with a DOI. The research data and parameters referenced in this study are well-documented in the associated published papers. All figures-source data (time arrays, mean curves, CI bands, pixel-wise $\Delta P_{\max}$ means and CIs) and scripts that generated them are available at [**GitHub https://github.com/waqashaseeb/nmqsd-vibronic-sse**].

4) Presentation and English

4.1 Typos and wording

"Line 220: 'protien' → 'protein'."
"Line 610: 'both case exhibit' → 'both cases exhibit'."

"Line 790: 'negative cohereces' → 'negative coherences'."
"Line 850: 'Futhermore' → 'Furthermore'."

Response: All corrected.

4.2 Clarity and units

"Throughout Results and Discussion: Sentences are often long and complex (e.g., Line ∼270–290). Please simplify for clarity. Notation: Units are inconsistently reported (cm^{-1} vs eV). Please provide conversions consistently and define all symbols (γ, γ_i, γ_{iE}) on first use."

Response: We shortened and clarified the identified passages, moved technical details to Methods, and added signposts for readability. We report energies in **eV** in figures and give a single conversion in Methods (1 eV = 8065.54 cm^{-1}). Symbols are defined at first use: ϵ (bias), Δ (electronic coupling), ω (mode frequency), γ_E (environmental coupling in the Itô diffusion), γ_i (Spike mode–ACE2 coupling), L (Lindblad operator), Population (ρ_{00}), and $\kappa(t)$ (instantaneous rate).

All updates are visible in the revised reference list and highlighted in orange color in the tracked-changes version.

Reviewer #3 :
We would like to express our sincere gratitude for your thorough review.

We appreciate the reviewers guidance; the manuscript is stronger and clearer as a result of these revisions. We are happy to move sensitivity results between the main text and SI or to adjust CI visualizations as preferred.

We are grateful for the opportunity to improve our work based on your expert critique and hope that you will find our revisions satisfactory. We look forward to your further guidance and the possibility of our study being published. Thank you once again for your constructive feedback and for considering our manuscript for publication.

Sincerely,
Mohamad Toutounji and Muhammad Waqas Haseeb
