# Supplementary material for: Non-markovian electron tunneling in SARS-CoV-2 virus infection in structured environments
Source: PLoS One. 2026 Apr 3;21(4):e0344447. doi: 10.1371/journal.pone.0344447 (PMC13048415; doi:10.1371/journal.pone.0344447)
Supplement: Attachment — Submitted filename: Response_to_Reviewers_PLOS.pdf [file pone.0344447.s006.pdf]

Response to Reviewers

Submission ID:[PONE-D-25-45999] - [EMID:88623151bdfdb171]

Manuscript Tittle: Non-Markovian Electron Tunneling in SARS-CoV-2 virus infection in Structured Environments.

Dear Editor and Reviewers,

We thank you for the careful reading and constructive suggestions. We have revised the manuscript, figures, and code accordingly. Below we respond point-by-point, quoting each comment in bold and then providing our response. Where helpful, we include exact text to be inserted into the manuscript and note figure/code changes.
Reviewer #1 :

We would like to express our sincere gratitude for your thorough review and insightful comments on our manuscript. Your feedback has been invaluable in guiding us to refine and enhance our study, ensuring that the final version of our paper is both robust and comprehensive. In response to your observations and suggestions, we have carefully revised our references.

In the following sections of this response letter, we detail the modifications in the references and additions we have made to our manuscript in light of your feedback.

Please make the following correction: References 3, 13, 26, and 29 are old, please use new references.

Response: We thank the reviewer for this valuable suggestion. We have systematically updated the bibliography throughout the manuscript. All items flagged as "old" (Refs. 3, 13, 26, 29,) were reviewed and, where appropriate, *replaced with recent peer-reviewed sources* from the last few years.

All updates are visible in the revised reference list and highlighted in orange color in the tracked-changes version.

We are grateful for the opportunity to improve our work based on your expert critique and hope that you will find our revisions satisfactory. We look forward to your further guidance and the possibility of our study being published. Thank you once again for your constructive feedback and for considering our manuscript for publication.

Sincerely,
Mohamad Toutounji and Muhammad Waqas Haseeb
